# Animations designed to raise patient awareness of prudent antibiotic use: patient recall of key messages and their immediate effect on patient attitude

**DOI:** 10.1186/s13104-017-3048-0

**Published:** 2017-12-06

**Authors:** Donna M. Lecky, Harpal Dhillon, Neville Q. Verlander, Cliodna A. M. McNulty

**Affiliations:** 1 Public Health England, Primary Care Unit, Microbiology Department, Gloucester Royal Hospital, Great Western Road, Gloucester, GL1 3NN England, UK; 2 Present Address: Shire, 1 Kingdon Street, London, W2 6BD England, UK; 3 Public Health England, Statistics and Modelling Economics Unit, 61 Colindale Avenue, London, NW9 5EQ England, UK

**Keywords:** Antibiotics, Primary care, Prevention, Patient education, Public health

## Abstract

**Objectives:**

This study aimed to determine if patients recalled key messages from antibiotic animations shown on digital displays in General Practice waiting rooms, and if watching them changed patients’ immediate intentions to consult their GP for upper respiratory tract infections, seek antibiotics and self-care.

**Results:**

The pre intervention focus group found the animations intergenerational, informative and educational. 3119 patients were observed in 3 GP practices during project team visits; 145 (4.6%) were observed watching the animations; 132 (91%) remembered seeing them; the key messages were retained by 47–55% of patients. Significant positive differences were observed for questions related to intended antibiotic related behaviours.

**Electronic supplementary material:**

The online version of this article (10.1186/s13104-017-3048-0) contains supplementary material, which is available to authorized users.

## Introduction

The increase in antibiotic resistant bacteria in recent years has had much publicity however despite this, many people often still expect antibiotics for self-limiting respiratory tract infections [1]. As patients are the end users of antibiotics their antibiotic use intentions are essential in any attempt to control antibiotic use and resistance.

To support the England antibiotic awareness campaign in 2012 a series of animations on prudent antibiotic use were developed to be displayed in GP practices, via the Life Channel network. In order to facilitate potential behaviour change the animations have a predefined target audience and the resources were developed especially for this group; [2] they contain empowering positive messages which have been identified as having a stronger influence on behaviour change; [3, 4] the use of a variety of animated animal characters appeals to different people however the message remains consistent in each animation; [2, 3] and the messages themselves are simple, clear and concise [4] and tailored to the literacy levels of the general public [5].

This study aimed to identify if these animations increased patient awareness of appropriate antibiotic use by assessing patient recall of the key messages from the animations and whether patient’s immediate intention to use antibiotics changed as a result of seeing the animations in GP practice waiting rooms.

## Main text

### Methods

#### Animation development

The antibiotic awareness animations were commissioned by the Department of Health and developed by a private company, Life Channel in collaboration with the English European Antibiotic Awareness Day (EAAD) working group. Animation development was an iterative process involving input from members of the general public and the Advisory Committee on Antimicrobial Resistance and Healthcare Associated Infections (ARHAI) public education subgroup. Care was taken to ensure that key messages were in line with National Guidance and Public Health campaigns at that time. The value of the animations were further discussed in 2012 with two focus groups (14 participants) of the general public recruited by the Public Health England People’s Panel, previously Health Protection Agency, who were not involved in the main study. Participants were shown each of the video clips and invited to discuss their views of the clips and how effective they might be in educating people about the appropriate use of antibiotics. Videos were modified accordingly prior to the main intervention.

#### Main intervention

**Fig. 1:**
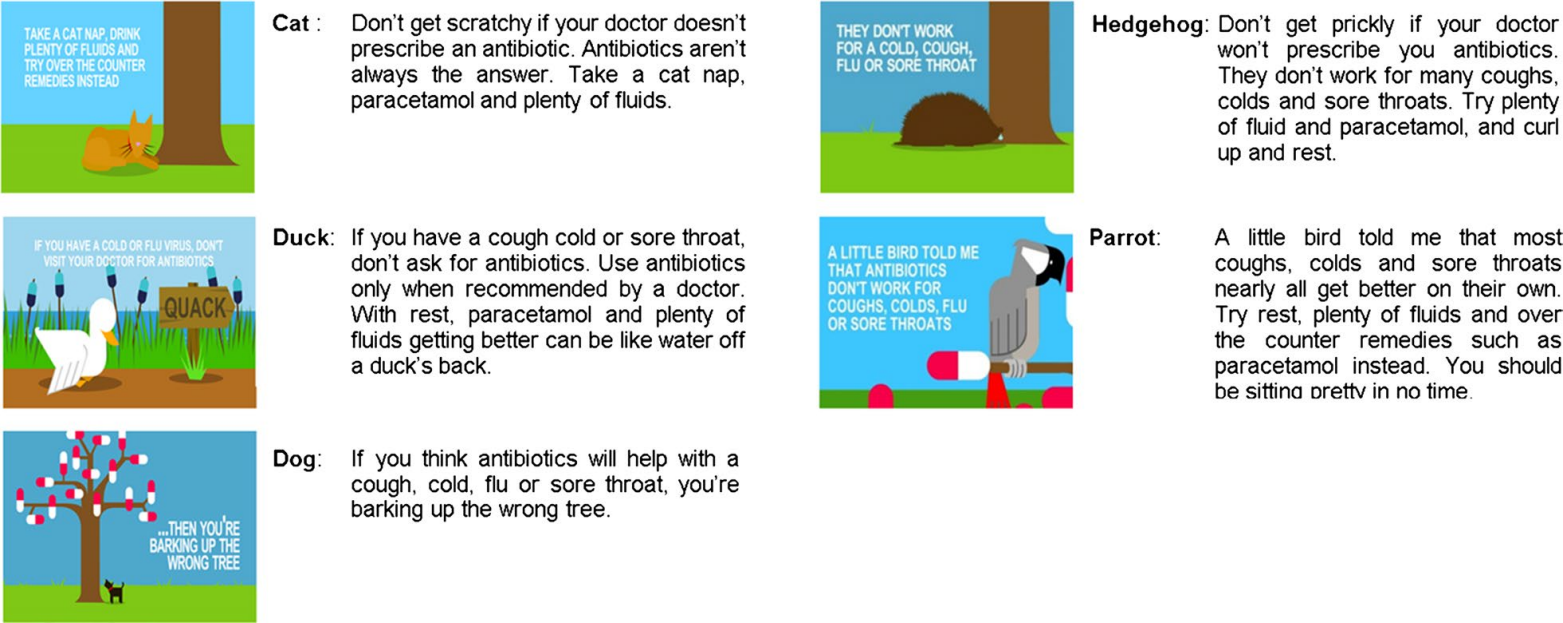
The animation scripts. Screen shots of each of the animations with associated animation text and dialogue

The animation series comprised of five 30 s long animations, each featuring different animals and key message (Fig. 1). GP practices within three primary care trusts (PCTs) in the West Midlands (Heart of Birmingham, Birmingham East and North and Walsall) were considered to participate if they subscribed to the Life Channel network [6], had a patient population ≥ 6000 in order to observe a constant patient footfall throughout the day with a low socioeconomic status demographic with the deprivation score: index of multiple deprivation [7] (IMD) 40.1–60 to include social grades C, D and E, i.e. those with low socioeconomic status, as this social grading has higher antibiotic use [8]. Eight eligible Practices were approached in random order until three agreed to participate.

The animations were aired from 16th November 2012, during the 2012 campaign, for 2 months. One of each of the animations was repeated every 20 min in each GP practice. A poster was on display during project team visits informing patients of the study. Each practice was visited by the project team 3–5 times during normal practice working hours who recorded how many patients were in the waiting area and how many could have watched the animations. Notes about the waiting area surroundings were also recorded. During a 4 h time period (12 animation viewings), a convenience sample of patients, over 16 years of age, who recalled seeing the animations were invited to complete a pre piloted short pictorial questionnaire (Additional file 1) in each Practice. Sample size was determined by how many participants were observed watching the animations during the allocated time period; researchers aimed for a minimum sample size of 130 participants across the study. As an aide memoire, screen shots of the animations were included in the questionnaire. The questionnaire comprised of a series of multiple choice questions on patient recall of the animations and their key messages and if the animations changed their attitudes towards antibiotics for cough, cold, flu or sore throat. There were seven change-of-attitude questions posed for each animation, of which three responses were permitted, namely “less likely”, “neutral” and “more likely”. We also asked patients if they themselves had a cough, cold, flu or sore throat in the past 6 months and if they requested antibiotics for any of these symptoms, in order to gauge baseline health seeking behaviour. Questionnaires were discussed with patients in the Practice waiting room prior to their GP consultation. The questionnaire went through numerous development iterations with input from the ARHAI public education subgroup. The questionnaire was then tested with other health professionals and the general public to ensure understanding and readability. Due to time constraints, a project officer asked patients the questions directly from the questionnaire and recorded their responses. Project teams did not engage in conversation with participants to prevent influencing their responses.

#### Data analysis

A binary response variable was created (“neutral”/”less or more likely”) and binomial probability test used to determine significant difference from 50%. Statistical significance level was chosen to be 5%. Statistical analysis was performed in STATA version 13.1.

### Results

#### Animation development: pre intervention focus group findings

Participants felt that the animal imagery was ‘intergenerational’, and that they could relate to the animal characteristics.

*‘The curling up of the hedgehog was good because if you are feeling unwell that’s what you intuitively do’*

and the familiar puns used: ‘Barking up the wrong tree’ and ‘quackery’. They felt that the animations were informative and educational by providing advice on alternatives to taking antibiotics, ‘Try rest, plenty of fluids and over the counter remedies such as paracetamol’,

*‘Leaves you in no doubt, that’s what you want [to do]’’*

and suggesting that people should not get annoyed if their GP refuses to prescribe antibiotics.

*The hedgehog clip makes reference that’s it’s not your doctor’s fault he is not prescribing you something you don’t need’*

#### Main intervention

*Practice observations* Project teams noted that the animations were not played once every 20 min or even at regular intervals. In some Practices, the TV was only switched on at the interviewer’s request; in one practice the patients could not see the TV screens as their seats faced in the opposite direction, this Practice was considered not eligible to participate. In some instances, a patient calling digital display was placed above the TV therefore interviewers were unsure if patients were actually watching the TV or watching to see if their name was called.

**Fig. 2:**
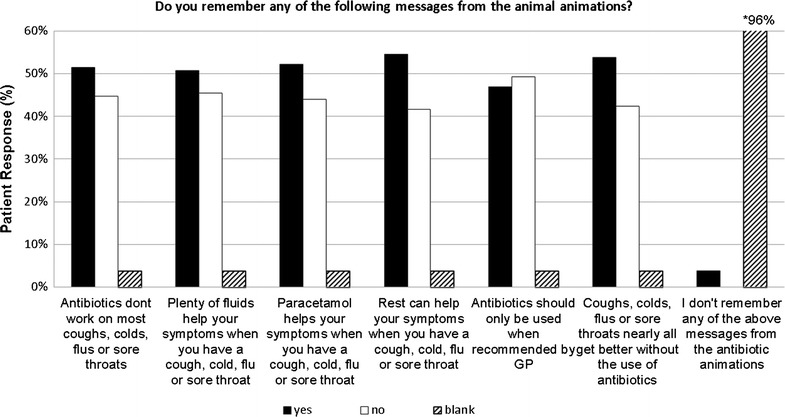
Patient recall of key messages after watching the antibiotic animations in GP practice waiting areas (n = 132). A bar chart representing percentage patient response to each of the ‘Do you remember any of the following messages from each of the animations?” questions

*Response rate* One hundred and forty-five of 3119 (4.6%) patients observed in the three practices appeared to be watching the animations and were approached to complete questionnaires. Of these, 132 (91%) reported seeing the animations and fully completed the questionnaires and comprised 74 (56%) male, 58 (44%) female; 116 (87%) were over 25 years old. The key messages of each animation were retained by 47–55% of patients (Fig. 2). Of patients who recorded having a cough, cold, sore throat or flu symptoms in the past 6 months, 75% (51/68) stated that they asked their GP for an antibiotic for these symptoms.

*Intention to change treatment behaviour (Table* *1* *)* Following the animation, a significant difference in patient response, towards positive intention to change behaviour, was observed for 5 of the 7 questions asked. Over half, 59.8% of respondents stated that watching the animation would make them less likely to see a GP the next time they had a cough, cold or sore throat (p = 0.001). Furthermore, 63% of respondents reported they would be less likely to “ask your GP for antibiotics the next time you have a flu, cough, cold or sore throat” (p < 0.001). More than half of patients (54.6%) reported that they would be more likely to drink plenty of fluids the next time they have a flu, cough, cold or sore throat (p = 0.01). Patients also reported that they were more likely to “rest the next time you have a flu, cough, cold or sore throat” and “take paracetamol the next time you have a flu, cough, cold or sore throat” (p < 0.001 and 65.9%, and p < 0.001 and 59.9%, respectively).

**Table 1:** Change in patients’ intention towards specific treatment options

Question Since waiting for your appointment today are you more or less likely to	Less likely (%)	Neutral (%)	More likely (%)	Not neutral	Difference to 50% neutral p-value
See your GP the next time you have a flu, cough, cold or sore throat (n = 132)	59.8	35.6	4.5	64.4% (95% CI 55.6, 72.5)	p = 0.001
Ask your GP for antibiotics the next time you have a flu, cough, cold or sore throat (n = 132)	62.9	31.8	5.3	68.2% (95% CI 59.5, 76.0)	p < 0.001
Drink plenty of fluids the next time you have a flu, cough, cold or sore throat (n = 132)	6.8	38.6	54.5	61.4% (595% CI 2.5, 69.7)	p = 0.01
Rest the next time you have a flu, cough, cold or sore throat (n = 132)	4.5	29.5	65.9	70.5% (95% CI 61.9, 78.1)	p < 0.001
Take paracetamol the next time you have a flu, cough, cold or sore throat (n = 132)	6.8	33.3	59.8	66.7% (95% CI 57.9, 74.6)	p < 0.001
Take antibiotics without the recommendation of a doctor or nurse (n = 131)	13.0	45.0	42.0	55.0% (95% CI 46.0, 63.4)	p = 0.3
Ask your GP for antibiotics the next time your child has a flu, cough, cold or sore throat (n = 129)	29.5	61.2*	9.3	38.8% (95% CI 30.3, 47.7)	p = 0.01

* Percentage neutral significantly different from 50% but not in a positive change of intention

There was no significant change in intention to “take antibiotics without the recommendation of a doctor or nurse” (p = 0.3 with 13% less likely and 42% more likely).

*Carers views* Only 29.5% of respondents with young children reported that watching the animations would make them less likely to ask their GP for antibiotics the next time their child (under 5 years of age) had a flu, cough, cold or sore throat” (p = 0.01) and 61.2% reported that they would be neither more or less likely.

### Discussion and conclusions

The very low rate of patients attending to the animations (4.6%) is surprising; it is possible that observers may have under-ascertained patient attention to the TV screen or patients were less likely to watch the animations due to issues previously outlined in Practice Observations section.

The evaluation examined patient’s intention to seek antibiotic therapy during their next episode or consultation; we did not examine whether the animations actually resulted in long term behaviour change as it has previously been identified that resources providing immediate and short-term benefits are of value [9]. Short-term attainable goals are also beneficial to help people succeed in the long term by guiding action in the here and how [10]. Influencing change in the adult population is difficult as their beliefs have been set and established over time. Our findings demonstrate that 60% of patients said their behaviour would change in the positive direction as a result of viewing the animations however, how well intentions translate into actual change varies [11, 12].

We found little difference in the intention to request antibiotics for a child after watching the animations. It has been suggested that parents are already quite knowledgeable regarding prudent antibiotic use and resistance [13], alternatively, it may be that parents trust their clinicians to decide what medication is appropriate for their children, and as such, the animations would not change their intended behaviour [14], or that the animations were not specifically targeted towards parents.

Our findings also demonstrated little change in patients’ response to taking non prescribed antibiotics, however 87% of our population were over 25 years old; other studies show that taking non prescribed antibiotics is uncommon in this group [8] so there may have been less opportunity for changing intentions in this area.

#### Implications

We have demonstrated that a simple and relatively inexpensive intervention was successful in positively influencing a patient’s immediate intention to use antibiotics although as a stand-alone resource, the impact may be less than the reach. However, as part of a multifaceted intervention to improve antibiotic use with a focus on reducing the risk of adverse health outcomes, [11] the animations could play a part in facilitating actual behaviour change. That being said, patients were often unable to view the animations due to screen placement suggests that any CCG commissioning this resource should audit their actual use and patient viewings to optimise their effectiveness within the primary care setting.

Our findings, i.e. the reach (4.6%) and effect of advertising/educational messages using commercial media outlets in GP waiting rooms, can be used to inform future research studies considering utilising this resource. Although there is often a variation between predicted future behaviour and actual future behaviour, our findings suggest that for a typical GP practice of 10,000 patients with 78,000 consults per year (300 consults/day for 260 days), 3588 (4.6%) patients would see the animations. With 60% less likely to consult or seek antibiotics the next time they have an RTI after watching the animations, this has the potential to equate to as many as 2153 less consultations per year.

## Limitations

- We do not know if the included practices are typical of other practices served by Life Channel.
- Long term behaviour change was not examined.

## Additional file

**Additional file 1.** The patient questionnaire.

## Abbreviations

EAAD: European Antibiotic Awareness Day
GP: general practitioner
IMD: index of multiple deprivation
MREC: Medical research Ethics Committee
PCT: primary care trust
RTI: respiratory tract infection
TV: television
