# Supplementary material for: Animations designed to raise patient awareness of prudent antibiotic use: patient recall of key messages and their immediate effect on patient attitude
Source: BMC Res Notes. 2017 Dec 6;10:701. doi: 10.1186/s13104-017-3048-0 (PMC5718068; doi:10.1186/s13104-017-3048-0)
Supplement: Supplementary file 1 — Additional file 1. The patient questionnaire. [file 13104_2017_3048_MOESM1_ESM.docx]

**Question 1**

At this point, how long have you been waiting in the surgery?

 Just arrived 5min or less  5-10 min  10 - 20 min  More than 20 min

**Question 2**

When was the last time you visited your surgery?

 In the last week  In the last fortnight  In the last month  More than a month ago

**Question 3**

**
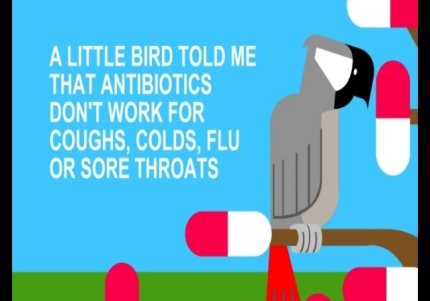

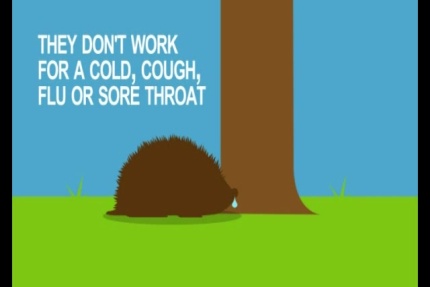

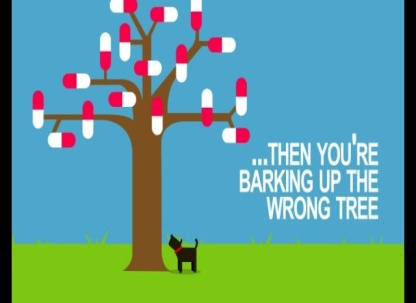

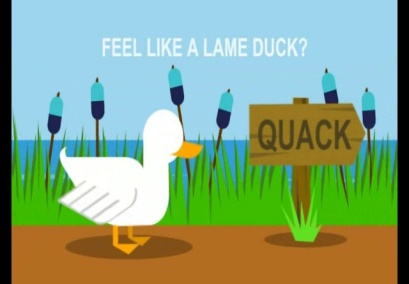

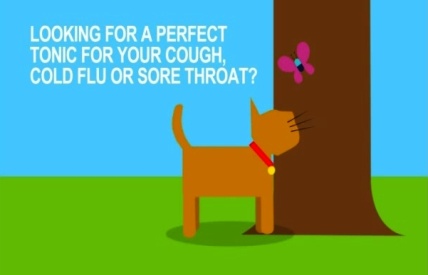
**Have you noticed videos featuring these animal characters *today or on previous visits to this surgery*?

 Yes  No

 Yes  No

 Yes  No

 Yes  No

 Yes  No

**Question 4**

Do you remember any of the following messages from the animal videos?

|  | **Yes** | **No** |
| --- | --- | --- |
| a) Antibiotics don’t work for most colds, coughs, flu or sore throat. |  |  |
| b) Plenty of fluids help your symptoms when you have a cough, cold, sore throat or flu. |  |  |
| c) Paracetamol helps your symptoms when you have a cough, cold, sore throat or flu. |  |  |
| d) Rest can help your symptoms when you have a cough, cold, sore throat or flu. |  |  |
| e) Antibiotics should only be used when recommended by a doctor. |  |  |
| f) Coughs, colds, flu, and sore throats nearly all get better on their own without antibiotics. |  |  |
| g) I don’t remember any of the above messages from the animal videos. |  |  |

**Question 5**

Since waiting for your appointment today are you now more or less likely to:

|  | **More Likely** | **Neutral** | **Less Likely** |
| --- | --- | --- | --- |
| a) See your GP the next time you have a flu, cough, cold or sore throat. |  |  |  |
| b) Ask your GP for antibiotics the next time you have a flu, cough, cold or sore throat. |  |  |  |
| c) Ask your GP for antibiotics the next time **your child** (under 5 years of age) has a flu, cough, cold or sore throat. |  |  |  |
| d) Take antibiotics without the recommendation of a doctor or nurse |  |  |  |
| e) Drink plenty of fluids the next time you have a flu, cough, cold or sore throat. |  |  |  |
| f) Rest the next time you have a flu, cough, cold or sore throat. |  |  |  |
| g) Take paracetomol (or other over the counter remedies) the next time you have a flu, cough, cold or sore throat. |  |  |  |

**Question 6**

Have you had a cough, cold, sore throat or flu symptoms in last 6 months?

 Yes  No  Don’t remember

**Question 7**

Have you asked your doctor for antibiotics in the last 6 months for a cough, cold, sore throat or flu symptoms?

 Yes  No  Don’t remember

**Question 8**

How (if at all) could the animal videos be improved to help the public request less antibiotics for coughs, colds, sore throats and flu symptoms?

 Yes  No  Don’t remember

**Question 9**

Are there any other comments you would like to make about the videos?
